# GLP-1 receptor regulates cell growth through regulating IDE expression level in Aβ1–42-treated PC12 cells

**DOI:** 10.1042/BSR20171284

**Published:** 2018-07-13

**Authors:** Huajie Li, Liping Cao, Yi Ren, Ying Jiang, Wei Xie, Dawen Li

**Affiliations:** Department of Neurology, the First People’s Hospital of Chang Zhou, Jiang Su 213003, China

**Keywords:** Alzheimer’s disease, GLP-1 receptor, IDE expression, type 2 diabetes

## Abstract

This study aimed to validate whether glucagon-like peptide-1 receptor (GLP-1R) / cyclic adenosine monophosphate (cAMP) / protein kinase (PKA) / insulin-degrading enzyme (IDE) signaling pathway was associated with neuronal apoptosis. We developed an animal model presenting both Alzheimer’s disease (AD) and type 2 diabetes (T2D), by crossing APP/PS1 mice (AD model) with streptozotocin (STZ)-treated mice (a T2D model). Neuronal apoptosis was detected by TUNEL staining and the expression levels of apoptosis-related proteins were examined by Western blotting. The viability of PC12 cells was analyzed by MTT assay and apoptosis of PC12 cells was detected by flow cytometry. The mRNA expression level was detected by qRT-PCR. T2D contributes to AD progress by prompting neuronal apoptosis and increasing expression of pro-apoptotic protein. β-Amyloid peptide1–42 (Aβ1–42) was shown to exert effects on inhibiting cell viability and prompting cell apoptosis of PC12 cells. However, GLP-1R agonist geniposide (Gen) significantly reversed them, exerting a protective role on PC12 cells. And IDE antagonist bacitracin (Bac) markedly reversed the protective effects of Gen on Aβ1–42-treated PC12 cells. Besides, Gen significantly reversed the effects of Aβ1–42 treatment on IDE expression, and the inhibitor of cAMP/PKA signaling pathway markedly reversed the effects of Gen on IDE expression level in Aβ1–42-treated PC12 cells. In conclusion, GLP-1R regulates cell growth, at least partially, through regulating cAMP/PKA/IDE signaling pathway in Aβ1–42-treated PC12 cells.

## Introduction

Alzheimer’s disease (AD) is an age-related neurodegenerative disorder, which is characterized by cholinergic neurodegeneration and progressive loss of memory [1]. There are two major pathological hallmarks of AD: neurofibrillary tangles formed by abnormal polymerization of microtubule-associated protein tau, and senile plaques composed of β-amyloid peptide1–42 (Aβ1–42) [2]. Increasing evidences demonstrated that type 2 diabetes (T2D) may play a relevant role in the development of AD [3–5]. Impaired insulin secretion and resistance was the relevant link between T2D and AD, and the glucose intolerance also seems to be associated with increased risk of AD [5].

Importantly, insulin-degrading enzyme (IDE), which is a 110 kDa Zn requiring metalloproteinase and is a major enzyme responsible for insulin degradation [6], has been proved to act as a junction point of T2D and AD [7]. In our previous study, we indicated that cyclic adenosine monophosphate (cAMP)/cAMP-dependent protein kinase (PKA) signaling pathway contributes to neuronal apoptosis via regulating IDE expression in a mixed model of T2D and AD [8].

Glucagon-like peptide-1 (GLP-1) is a glucoincretin hormone that plays important role in insulin-secreting β-cells, which lowers blood glucose and food intake, and enhances pancreatic islet β-cell proliferation and glucose-dependent insulin secretion in patients with T2D [9,10]. Importantly, cAMP/PKA signaling pathway is believed to be involved in the cellular mechanisms that mediate the inhibitory actions of GLP-1 on glucagon secretion, and GLP-1 receptor (GLP-1R) agonist was found to stimulate β-cell replication via activation of cAMP/PKA signaling pathway [11]. Besides, Wu et al. [12] indicated that human GLP-1 has the ability to activate the GLP-1R, induces PC12 cell differentiation, and has neurotrophic effects. Therefore, it raises the possibility that GLP-1R/cAMP/PKA/IDE signaling pathway may be associated with neuronal apoptosis, which may be one of the mechanisms underlying the linkage of IDE with T2D and AD.

Since neuronal loss or apoptosis is a vital contributor factor to AD progression, thus, we investigate the role of GLP-1R/cAMP/PKA/IDE signaling pathway on the growth of nerve cell PC12 in this study. Firstly, we showed that T2D contributed to AD progress by prompting neuronal apoptosis and increasing expression of pro-apoptotic protein. Aβ1–42 was used to inhibit cell viability and induce cell apoptosis of PC12 cells. GLP-1R agonist geniposide (Gen) was then used to validate its role on cell viability and apoptosis of Aβ1–42-treated PC12 cells. The effects of Gen on the expression of IDE in Aβ1–42-treated PC12 cells were detected by Western blotting. Moreover, H89, an inhibitor of cAMP/PKA/IDE signaling pathway were used, and the expression of IDE in Aβ1–42-treated PC12 cells was also detected.

## Methods

### Animals

The AD model was the double transgenic APPSwe/PS1 mice (B6C3-Tg (APPswe, PSEN1dE9) 85Dbo/ J), which were from Jackson Laboratories (Bar Harbor, Maine, USA). APPSwe is the Swedish mutation of the amyloid precursor protein, and PS1 is the mutant form of human presenilin 1 [13]. Animals were maintained at room temperature (25 ± 2°C) under a controlled environment in a 12 h light/12 h dark cycle, with free access to water and food.

### Induction of T2D mice

After fasting overnight, mice (age of 1.5 months) were received a single intraperitoneal injection of streptozotocin (STZ; 50 mg/kg; Sigma-Aldrich, St Louis, MO, USA) as previously described [14], which is widely used in the induction of T2D mice model [15]. STZ solution was prepared by dissolving it in 0.1 M citrate buffer (pH 5.5) and was terminally sterile-filtered. Mice with fasting blood glucose levels above 12 mM and higher insulin levels were considered T2D mice. The mice were killed (at age of 4 months) and brain samples were collected for analysis. Procedures were approved by the First People’s Hospital of Chang Zhou.

### TUNEL staining

The animals were anesthetized with pentobarbital sodium and perfused with 0.1 M phosphate-buffered saline (PBS, pH 7.4), followed by 4% paraformaldehyde in 0.1 M PB (pH 7.4). Brains were removed, post-fixed in the same fixative for 48 h, and dehydrated in alcohol. Slides were treated with xylene for 1 h and a graded series of ethanol and double distilled water washes; protease K incubation for 15–30 min at; 1% Triton X-100 washing for 8 min. The TUNEL staining was performed using the In Situ Cell Death Detection Kit, POD, following the manufacturer’s instructions (Roche Applied Science, Mannheim, Germany).The slides were analyzed with light microscopy.

### Cell culture and treatments

PC12 cells were obtained from Shanghai Institute for Cell Research, Chinese Academy of Sciences. The cells were grown in DMEM supplemented with 10% fetal bovine serum and 1% antibiotics (penicillin/streptomycin, Sigma-Aldrich, St Louis, MO, USA) in a humidified atmosphere of 95% air and 5% CO_2_ at 37°C. The medium was changed every 2–3 days. Aβ1–42 oligomers were obtained from Sigma (Sigma-Aldrich, St Louis, MO, USA) and were added to cell cultures at a final concentration of 0, 1.25, 2.5, 5, and 10 μM. Gen was obtained from the National Institute for the Control of Pharmaceutical and Biological Products and was added to cell cultures at a final concentration of 0, 0.1, 1.0, and 10 μM. For treatment with IDE antagonist bacitracin (Bac), PC12 cells were incubated with 1 mg/l Bac (Shanghai Huding technology Co., Ltd., China) for 30 min. For PKA inhibitor experiment, PC12 cells were treated with 20 μM H89 (Calbiochem, La Jolla, CA), which was prepared as a 4 mM stock solution in DMSO.

### Transfection

ShRNA-GLP-1R or Scramble shRNA were obtained from GeneChem (Shanghai, China). PC12 cells (1.0 × 10^6^) were transfected with 4 µg of shRNA-GLP-1R or Scramble shRNA by using Lipofectamine 2000.

### Cell viability assay

MTT assay was used to detect cell viability. PC12 cells (2 × 10^4^ per well) were seeded in 96-well plates for incubation overnight. PC12 cells were treated with various concentrations of exogenous substances for 24 h. Following incubation, 10 μL MTT (5 mg/ml) were added to each well for 4 h at 37°C. The culture medium was then removed and 100 μl DMSO was added to dissolve the formazan crystals. Absorbance was measured at 570 nm with an ELISA reader (Model680, Bio-Rad, USA) and cell viability was expressed as a percentage of the value against the non-treated control group.

### Flow cytometry

Cell apoptosis was detected by the flow cytometry analysis as described previously [16]. Cells were digested using trypsin and centrifuged at 200 ***g*** for 5 min. After washing, cells were resuspended, centrifuged and the pellet was resuspended in 1 ml NaCl/Pi. After an addition of DNase-free RNase A (Sigma-Aldrich, St Louis, MO, USA), cells were incubated at 37°C for 30 min. The propidium iodide (PI) was added and incubated at room temperature for 15 min, followed by transferred to Falcon tubes. By using a linear amplification in the FL-2 channel of a FACScan flow cytometer (Becton Dickinson, Rockville, MD, USA) equipped with cellquest software (Becton Dickinson), the number of apoptotic cells was measured.

### Western blotting

Western blotting were performed as previously described [17]. In brief, tissue samples were lysed in RIPA buffer containing 150 mM NaF, 2 mM sodium orthovanadate, and protease inhibitors (protease inhibitor mixture; Roche, Switzerland). Protein of total lysate (20 μg) was loaded and blotted. The membranes were incubated with primary antibodies anti-IDE (MMS-282R; 1:1000; Covance, UK), anti-cleaved caspase-3 (Cell Signaling, Danvers, MA, USA), anti-cleaved caspase-9 (STS, Cayman Chemical, Michigan, USA), and anti-cleaved caspase-8 (Cell Signaling, Danvers, MA, USA) overnight at 4°C, and then reacted with HRP-conjugated secondary antibodies (1:1000; Santa Cruz Company, CA, USA) at room temperature for 1.5 h. The protein bands were detected by ECL and visualized by UVP Gel imaging system (Upland, CA). The band intensity was analyzed by AlphaEaseFC (version 4.0). GAPDH served as the loading control.

### Quantitative real-time RT-PCR

RNA was extracted from the frozen right hippocampus using Trizol reagent (Invitrogen, Life Technologies, CA, USA). RNA was quantified using a NanoDrop spectrophotometer (Thermo Scientific, USA). The cDNA templates were synthesized with the SuperScript III First-Strand Synthesis SuperMix. The following oligonucleotide sequences were used as primers: IDE, 5′-CAATACATTCAGAAGCTACGTG-3′ (forward) and 5′-CAGGGTATGGTGTTGCATCTT-3′ (reverse). GAPDH, 5′-CATCACCATCTTCCAGGAGCG-3′ (forward) and 5′-TGACCTTGCCCA CAGCCTTG-3′. Real-time RT-PCR was performed by using a Taq-Man gene expression assay kit (Invitrogen, Life Technologies, CA, USA).

### Statistics

Data were analyzed using the program Prism (GraphPad Software, Inc., La Jolla, CA, USA). Data were expressed as means ± SEM. Data were analyzed by one-way or two-way ANOVA. Statistical significance was set as *P*<0.05.

## Results

### Neuronal apoptosis in mice with T2D and AD model mice

**Figure 1:**
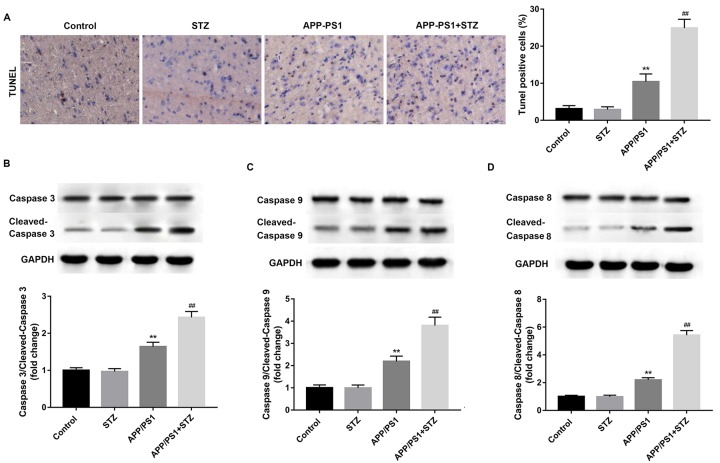
Neuronal apoptosis in mice with T2D and AD model mice APP/PS1 mice served as AD model, and STZ-treated mice served as T2D model. (**A**) The apoptotic cell in T2D and AD mice (APP/PS1+STZ) was significantly increased as compared with the AD mice (APP/PS1). The expression levels of cleaved caspase-3 (**B**), -9 (**C**), and -8 (**D**) in T2D and AD mice were significantly increased as compared with the AD mice. GAPDH served as the loading control. **P<0.01 vs. Control group, and ^##^*P*<0.01 vs. APP/PS1 group. *n* = 15 for each group.

We developed an animal model presenting both AD and T2D, by crossing APP/PS1 mice (AD model) with STZ-treated mice (T2D model). Neuronal apoptosis and the expression levels of apoptosis-related proteins were detected in the isolated hippocampus tissues. As shown in Figure 1, no significant difference was found in STZ-treated and control mice. The apoptotic cell in AD mice was greatly increased as compared with the control mice, and the apoptotic cell in T2D and AD mice was dramatically increased as compared with the AD mice (Figure 1A). No significant differences in the expression levels of cleaved caspase-3, -9, and -8 were found in STZ-treated and control mice. However, the expression levels of cleaved caspase-3, -9, and -8 in AD mice were significantly increased as compared with the control mice, and the expression levels of cleaved caspase-3, -9, and -8 in T2D and AD mice were significantly increased as compared with the AD mice (Figure 1B–D). These results may indicate that T2D contributes to AD progress by prompting neuronal apoptosis.

### GLP-1R agonist Gen reversed the effects of Aβ1–42 treatment on cell viability and apoptosis of PC12 cells

**Figure 2:**
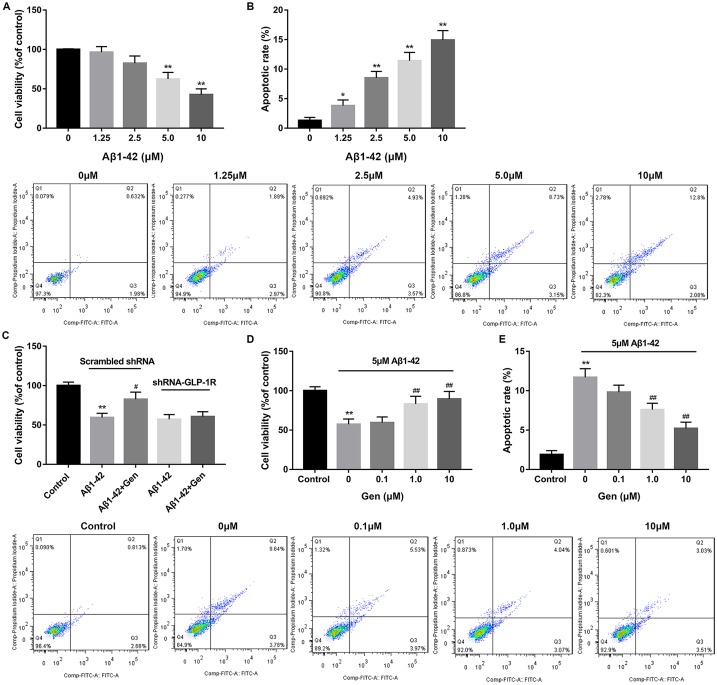
GLP-1R agonist Gen reversed the effects of Aβ1–42 treatment on cell viability and apoptosis of PC12 cells (**A**) Aβ1–42 treatment significantly inhibited cell viability of PC12 cells as compared with the control. (**B**) Aβ1–42 treatment significantly induced cell apoptosis of PC12 cells as compared with the control. (**C**) GLP-1R knockdown decreased the protective role of Gen (1 μM) on PC12 cells. (**D**) GLP-1R agonist Gen reversed the effects of Aβ1–42 treatment on cell viability of PC12 cells. (**E**) GLP-1R agonist Gen reversed the effects of Aβ1–42 treatment on cell apoptosis of PC12 cells. *P<0.05, **P<0.01 vs. 0 μM Aβ1–42 or Control group, ^#^*P*<0.05 vs. Aβ1–42 treatment alone group, and ^##^*P*<0.01 vs. 0 μm Gen group.

Our findings mentioned above implicated an important role of neuronal apoptosis in T2D the AD model. Therefore, on this basis, neuronal cells PC12 were used to further explore how neural function is regulated or controlled *in vitro* by T2D- the AD-related factors, such as Aβ1–42 and GLP-1R. Data revealed that Aβ1–42 treatment effectively inhibited cell viability of PC12 cells in a dose-dependent manner as compared with the control (Figure 2A). In contrast, Aβ1–42 treatment markedly induced cell apoptosis of PC12 cells in a dose-dependent manner in comparison with the control (Figure 2B). After that, a dose of 5 μM Aβ1–42 was used for the following study.

To validate the specificity of GLP-1R agonist Gen, GLP-1R was knocked down and transfected into PC12 cells. The results showed that, 5 μM Aβ1–42 significantly inhibited cell viability of PC12 cells as compared with the control. However, 1 μM Gen significantly increased cell viability of Aβ1–42-treated PC12 cells, exerting a protective role on PC12 cells. after GLP-1R knockdown, the protective role of Gen on PC12 cells was abolished (Figure 2C). After treatment of Gen with different concentrations, we found that the cell viability of Aβ1–42-treated PC12 cells was significantly increased in a dose-dependent manner, which markedly reversed the effects of Aβ1–42 on cell viability (Figure 2D). Gen also inhibited cell apoptosis of Aβ1–42-treated PC12 cells in a dose-dependent manner, and effectively reversed the effects of Aβ1–42 on cell apoptosis (Figure 2E).

### IDE antagonist Bac reversed the effects of Gen on the cell viability and apoptosis of Aβ1–42-treated PC12 cells

**Figure 3:**
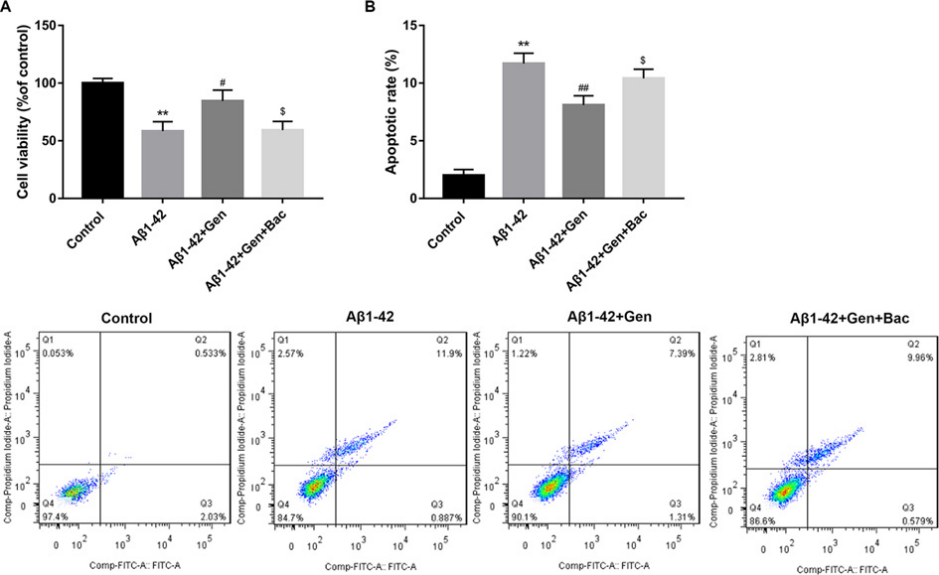
IDE antagonist Bac reversed the effects of Gen on the cell viability and apoptosis of Aβ1–42-treated PC12 cells (**A**) IDE antagonist Bac significantly reversed the effects of Gen on the cell viability of Aβ1–42-treated PC12 cells. (**B**) IDE antagonist Bac significantly reversed the effects of Gen on the cell apoptosis of Aβ1–42-treated PC12 cells. **P<0.01 vs. Control group, ^#^*P*<0.05, ^##^*P*<0.01 vs. Aβ1–42 treatment alone group, and ^$^*P*<0.05 vs. Aβ1–42+Gen group.

Further study showed that IDE antagonist Bac significantly reversed the effects of Gen on the cell viability of Aβ1–42-treated PC12 cells, as evidenced by the marked decreased cell viability in the Aβ1–42+Gen+Bac group as compared with the Aβ1–42+Gen group (Figure 3A). Besides, IDE antagonist Bac significantly reversed the effects of Gen on the cell apoptosis of Aβ1–42-treated PC12 cells, as evidenced by the marked increased cell apoptosis in the Aβ1–42+Gen+Bac group as compared with the Aβ1–42+Gen group (Figure 3B).

### Inhibitor of cAMP/PKA signaling pathway reversed the effects of Gen on the expression of IDE in Aβ1–42-treated PC12 cells

**Figure 4:**
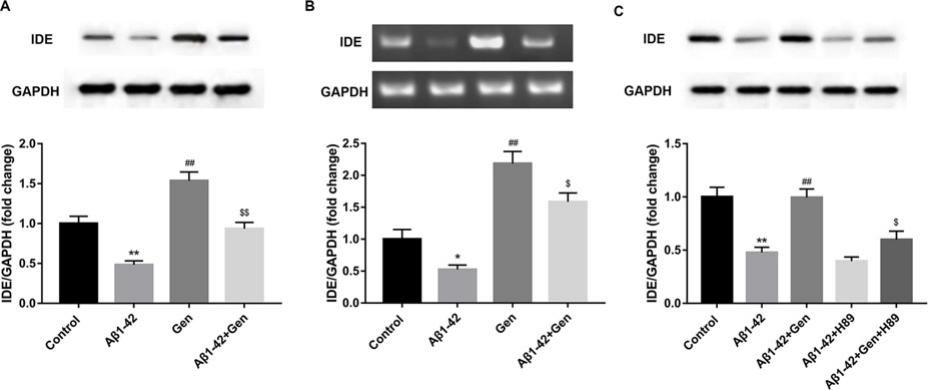
Inhibitor of cAMP/PKA signaling pathway reversed the effects of Gen on the expression of IDE in Aβ1–42-treated PC12 cells (**A**) Aβ1–42 treatment significantly decreased the protein level of IDE as compared with the control, and Gen significantly reversed the effects of Aβ1–42. (**B**) Aβ1–42 treatment significantly decreased the mRNA level of IDE as compared with the control, and Gen significantly reversed the effects of Aβ1–42. (**C**) PKA inhibitor H89 treatment significantly reversed the effects of Gen on the expression of IDE in Aβ1–42-treated PC12 cells. GAPDH served as the loading control. *P<0.05, **P<0.01 *vs.* Control group, ^##^*P*<0.01 *vs.* Aβ1–42 treatment alone group, and ^$^*P*<0.05, ^$$^*P*<0.01, *vs.* Gen or Aβ1–42+Gen group. *n* = 15 for each group.

We found that Aβ1–42 treatment significantly decreased the protein level of IDE as compared with the control, and Gen significantly reversed the effects of Aβ1–42. Indeed, we found that Gen treatment alone significantly increased the protein level of IDE as compared with the control (Figure 4A). Consistently, the mRNA level of IDE showed similar expression pattern (Figure 4B). Treatment of PKA inhibitor H89 significantly reversed the effects of Gen on the expression of IDE in Aβ1–42-treated PC12 cells, as evidenced by the markedly decreased protein expression level of IDE ac compared with the Aβ1–42+ Gen group (Figure 4C).

## Discussion

The death of neurons is one of the hallmarks of AD, at least, some of the functional impairments in AD are likely due to the death of neurons or the processes that ultimately lead to the death [18]. Thus, studies of the molecular mechanisms by which neurons or other cell types die are of potential importance to this disease. Dysfunction of neuronal survival signaling pathway also occurs in neurons in T2D patients. In the STZ-induced T2D rat model, the hippocampus of rats showed decreased expression levels of neuronal survival factors such as insulin-like growth factor 1 receptor a (IGF-Ira), protein kinase B (Akt/PKB), and cAMP response element-binding protein (CREB) [19]. In our study, we found that the apoptotic cell in T2D and AD mice was significantly increased as compared with the AD mice. The expression levels of cleaved caspase-3, -9, and -8 in T2D and AD mice was significantly increased as compared with the AD mice. These results may indicate that T2D contributes to AD progress by prompting neuronal apoptosis. The above-mentioned results were further confirmed by our previous findings that T2D contributes to the AD progress by accelerating and worsening spatial memory and recognition dysfunctions examined by morris water maze test and recognition task [8].

Evidences have revealed the influence of GLP-1 in the nervous system. In some studies on neurocytology and electrophysiology, GLP-1 has been found to play an important role as an anti-amyloid, preventing long-term potentiation (LTP) from amyloid injury and promoting LTP [9,20,21]. GLP-1 and GLP-1R were summarized to result in glucose-dependent insulin secretion, induction of cell proliferation, and enhanced resistance to apoptosis [11]. In our study, we found that GLP-1R agonist Gen reversed the effects of Aβ1–42 treatment on cell viability and apoptosis of PC12 cells. These results showed that GLP-1R has cytoprotective effect on PC12 cells by enhancing cell viability and inhibiting cell apoptosis.

Previous study indicated that GLP-1R agonists have cytoprotective effect on β cells by regulating proliferative, differentiation/neogenesis, and apoptosis [11]. The underlying mechanism was associated with activation of cAMP/PKA with subsequent phosphorylation and activation of CREB [22,23]. cAMP/PKA signaling pathway is a potential therapeutic treatment of T2D due to its regulative role in glucose homeostasis by modulating glucagon and insulin secretion, glycogen synthesis, gluconeogenesis, glucose uptake, and breakdown and neural control of glucose homeostasis [24]. Besides, it has long been known that cAMP/PKA signaling pathway also plays an important role in neuronal survival and in the formation of long-term memory [25,26]. Besides, the impairment of the cAMP/PKA pathway in AD has mainly been attributed to Aβ toxicity [27].

Our previous study suggested that cAMP/PKA signaling pathway contributed to neuronal apoptosis via regulating IDE expression in a mixed model of T2D and AD [8]. In our study, we found that Aβ1–42 treatment significantly decreased the protein level of IDE as compared with the control, and Gen significantly reversed the effects of Aβ1–42. Previous results showed that Gen induced the expression of IDE in a dose-dependent manner. Moreover, Bac, an inhibitor of IDE, and RNAi on GLP-1R gene decreased the neuroprotection of Gen in Aβ1–42-treated cortical neurons [28]. Our study indicated that IDE antagonist Bac reversed the effects of Gen on the cell viability and apoptosis of Aβ1–42-treated PC12 cells. Besides, H89, an inhibitor of cAMP/PKA signaling pathway reversed the effects of Gen on the expression of IDE in Aβ1–42-treated PC12 cells. These investigations indicated that GLP-1R could regulate IDE expression by modulating cAMP/PKA signaling pathway.

IDE acts as a junction point of T2D and AD [7]. On one hand, IDE expression is strongly associated with classic features of T2D-hyperinsulinemia, decreased insulin degradation, and glucose intolerance [29]. Moreover, Bac could increase amyloid accumulation in the culture of pancreatic β-cells [30]. These results demonstrate that the regulation of the IDE level and activity may contribute to the T2D pathogenesis. On the other hand, in the brain, IDE is secreted from microglial cells and neurons, and degrades Aβ extracellularly and on the cell surface [31,32]. Despite these investigations, the mechanism underlying the linkage of IDE with T2D and AD remains unclear. The conclusion of this study that GLP-1R/cAMP/PKA/IDE signaling pathway may be associated with neuronal apoptosis, might be one of the mechanisms underlying the linkage of IDE with T2D and AD.

To sum up, our study showed that GLP-1 receptor regulates cell growth, at least partially, through regulating cAMP/PKA/IDE signaling pathway in Aβ1–42-treated PC12 cells.

## Abbreviations

Aβ1–42: β-amyloid peptide1–42
AD: Alzheimer’s disease
cAMP: cyclic adenosine monophosphate
CREB: cAMP response element-binding protein
GLP-1R: glucagon-like peptide-1 receptor
IDE: insulin-degrading enzyme
PKA: protein kinase
STZ: streptozotocin
T2D: type 2 diabetes
